# Genetic programs controlling cortical interneuron fate

**DOI:** 10.1016/j.conb.2013.12.012

**Published:** 2014-06

**Authors:** Nicoletta Kessaris, Lorenza Magno, Anna Noren Rubin, Marcio Guiomar Oliveira

**Affiliations:** Wolfson Institute for Biomedical Research and Department of Cell and Developmental Biology, University College London, Gower Street, London, WC1E 6BT, UK

## Abstract

The origins of cortical interneurons in rodents have been localized to the embryonic subcortical telencephalon where distinct neuroepithelial precursors generate defined interneuron subsets. A swathe of research activity aimed at identifying molecular determinants of subtype identity has uncovered a number of transcription factors that function at different stages of interneuron development. Pathways that lead to the acquisition of mature interneuron traits are therefore beginning to emerge. As genetic programs are influenced by external factors the search continues not only into genetic determinants but also extrinsic influences and the interplay between the two in cell fate specification.

## Highlights

• Cortical interneurons originate in the embryonic subcortical telencephalon.
• Spatial and temporal control of progenitor differentiation generates diversity.
• Genetic pathways of interneuron cell fate specification.
• Intrinsic pathways and extrinsic cues interplay in interneuron specification.

**Current Opinion in Neurobiology** 2014, **26**:79–87

This review comes from a themed issue on **Inhibition: synapses, neurons and circuits**

Edited by **Gordon Fishell** and **Gábor Tamás**

For a complete overview see the Issue and the Editorial

Available online 15th January 2014

0959-4388/$ – see front matter, © 2014 Elsevier Ltd. All rights reserved.

**http://dx.doi.org/10.1016/j.conb.2013.12.012**

## Introduction

GABAergic interneurons constitute 20–30% of all neurons in the cortex and are essential for cortical circuit function. Through their inhibitory actions cortical interneurons have multiple functions including maintenance of network balance and shaping of synchronized activity [1]. This functional diversity of interneurons in the cortex is enabled through a remarkable heterogeneity. The exact number of different subtypes that exist in the adult cortex is unclear partly because of ambiguity in their classification. However, recent concerted efforts to pull together different criteria provide great promise for a unifying classification scheme [2,3^••^].

Tremendous efforts have been made in the last 15 years to determine how interneuron heterogeneity becomes established (recently reviewed in [4,5^••^,6,7^••^,8]). It is now widely accepted that genetic pathways hold the key to cell fate determination. Insight into the genetics that drive cell diversity is emerging fast and has already had far reaching benefits beyond basic science into neurodevelopmental disease research and stem cell therapies [9,10^••^,11]. In this review we describe the known genetic regulatory pathways that promote cortical interneuron cell fate specification focusing mainly on the most recent advances in the field. As intrinsic genetic programs of cell identity do not act in isolation, we discuss how extrinsic cues influence the development of cortical interneurons.

## Generating interneuron diversity

**Figure 1:**
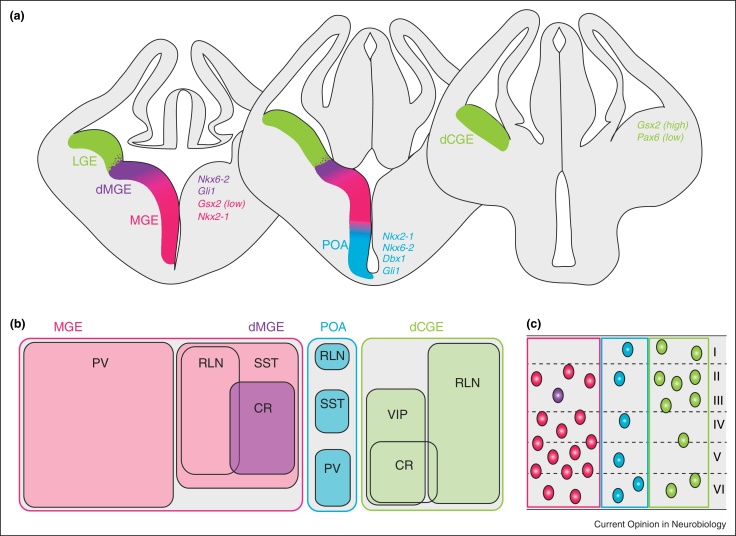
Embryonic origin and mature fates of cortical interneurons. **(a)** Schematic showing the subdivisions of the embryonic telencephalon. The three regions where cortical and hippocampal interneurons originate are the medial ganglionic eminence (MGE) (including the dorsal MGE-dMGE), the caudal ganglionic eminence (CGE) and the preoptic area (POA). The dorsal part of the CGE (dCGE) is a caudal extension of the lateral ganglionic eminence (LGE) and is distinct from the MGE. These progenitor zones of the telencephalon can be identified by combinatorial expression of transcription factors within the neuroepithelium. **(b)** The major classes of cortical interneurons that originate from the three neuroepithelial regions during embryogenesis can be identified using neurochemical markers. The MGE generates 60% of all cortical interneurons and includes mainly parvalbumin (PV)-expressing and somatostatin (SST)-expressing subtypes. A large fraction of the SST cells co-express reelin (RLN). SST/calretinin (CR) co-expressing interneurons are derived exclusively from the dMGE. The POA generates ∼10% of all cortical interneurons and includes a variety of subtypes. The dCGE is the source of ∼30% of all cortical interneurons. Half of these co-express RLN and smaller fractions express vasoactive intestinal peptide (VIP) and/or CR. Note that neuropeptide Y (not shown in the figure) is also expressed in subsets of cortical interneurons, including ones derived from the POA. **(c)** Distribution of MGE (red)-derived, POA (blue)-derived and dCGE (green)-derived interneuron populations within the different layers (I–VI) of the adult cortex.

The generation of interneuron diversity begins during embryogenesis when cortical and hippocampal interneurons are born in subcortical regions and migrate away to reach their final positions. Three sources of cortical interneurons have been identified in the telencephalon: the medial ganglionic eminence (MGE), the caudal ganglionic eminence (CGE) and the preoptic area (POA) (Figure 1). Each of these regions generates distinct cohorts of interneurons for the cortex indicating that restriction of neurogenic potential in the subpallium generates diversity (Figure 1).

The three sources of interneurons identified to date are clearly not enough to explain the >20 subtypes of mature interneurons found in the adult cortex and hippocampus [1,12]. Original suggestions that the septum — the fourth major germinal zone of the ventral telencephalon — may generate interneurons for the cortex have been disproved [13]. However, smaller subdivisions of the neuroepithelium lining the ganglionic eminences have been identified based on transcription factor expression, raising the possibility that finer restriction of neurogenic fate from the three major sources may contribute to diversity [14]. In agreement with this, biases in interneuron subtype generation have been described within the ganglionic eminences and the POA along the dorso-ventral and anterior–posterior axes (see dMGE and POA sections below) [4,5^••^,6,7^••^,8].

Superimposed on the spatial control of interneuron fate is temporal regulation, with distinct interneurons being generated at different stages during development [15,16,17^••^]. The temporal regulation of cell identity within the MGE has recently been attributed partly to the presence of distinct precursors for upper and lower layer MGE-derived interneurons [18^••^]. One question that ensues is whether committed precursors of early-born and late-born interneurons in the ganglionic eminences are intermingled but molecularly distinct from each other, as recently shown for pyramidal neuron precursors [19].

## Spatial genetic patterning of the neuroepithelium and initiation of the cortical interneuron development pathway

**Figure 2:**
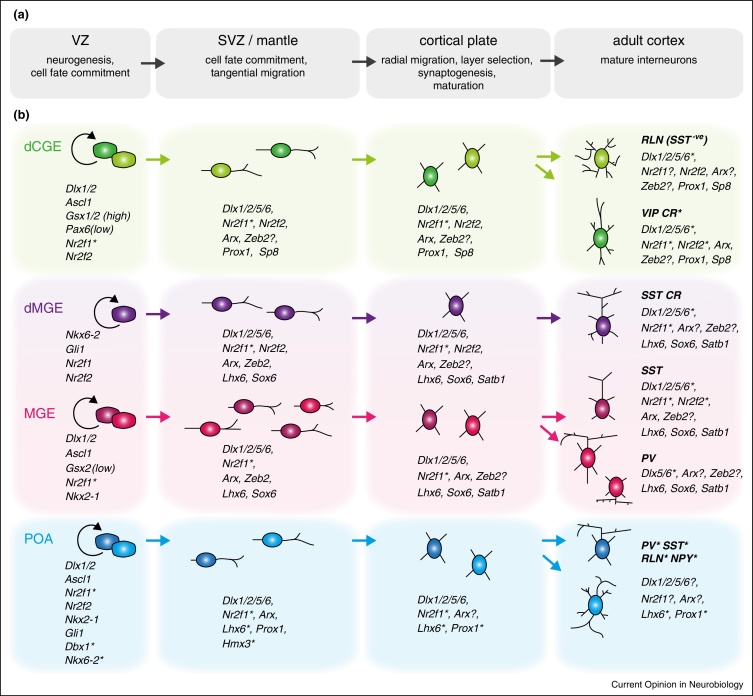
Genetic programs controlling cortical interneuron development. **(a)** Progressive stages of cortical interneuron development. **(b)** Cortical interneuron development from the three major telencephalic sources: the MGE, the dCGE and the POA. Transcription factors involved at different stages of cortical interneuron development are shown. Some of these factors participate broadly in interneuron development (e.g. members of the DLX and NR2F families and ARX). ZEB2 has been described in the MGE lineage but may also be expressed in other interneuron populations. Other transcription factors are unique to specific domains and/or stages of differentiation: NKX2-1 defines the MGE neuroepithelium and activates a cascade of genes downstream including *Lhx6*, *Sox6* and *Satb1*; NKX6-2 and GLI1 are enriched in the neuroepithelium of the dMGE (although not restricted to that region) and provide this domain with its unique identity and differentiation potential; DBX1 and HMX3 have been used to fate-map the POA because of their restricted expression in this domain; PROX1 and SP8 have been identified as being expressed in CGE-derived cortical interneurons at all stages of their development. Although depicted as having common precursors, interneurons that originate from the same neuroepithelial domain may arise from lineages that split early during development. Note that the VZ of the dMGE expresses MGE transcription factors in addition to the dMGE-specific genes indicated. ? indicates that expression is unclear or unknown. * indicates expression in some but not all cells. Expression of *Zeb2*, *Sox6* and *Satb1* has not been examined in POA-derived cortical interneurons.

Much like the spinal cord where morphogen-regulated transcription factors establish distinct progenitor domains [20], the telencephalic subdivisions arise through the activation of transcription factors that provide the neuroepithelial cells with their identity (Figures 1 and 2). Morphogens that pattern the telencephalon include SHH and FGF and early-acting transcription factors include GLI1/2/3, PAX6, SIX3, FOXG1, NKX2-1, GSX2, ASCL1 and NEUROG2 [21–24]. These transcription factors function well before the appearance of any cortical interneurons and yet have profound effects on cortical interneuron development through restriction of progenitor differentiation potential.

At the top of the genetic cascade of cortical interneuron development are the transcription factors DLX1 and DLX2 which are activated in all interneurons downstream of early patterning genes (Table 1 and Figure 2). DLX1/2 have multiple roles at the initial stages of cortical interneuron development including inhibition of glial fate, promotion of GABAergic differentiation and cell migration [4,5^••^,6,7^••^,8]. ARX and DLX5/6 are two direct targets of DLX1/2. They are transcription factors that show prolonged expression in subsets of cortical interneurons beyond the initial specification and migration stages and are deployed in multiple ways in the regulation of interneuron development [4,5^••^,6,7^••^,8].

ASCL1 is another transcription factor that is expressed in the subcortical telencephalon and is thought to function high up in the hierarchy of cortical interneuron development. ASCL1 loss-of-function (LOF) mutants have implicated this factor in the regulation of neurogenic differentiation genes [23]. More recent compound DLX1/2 and ASCL1 LOF mouse mutants have revealed unique and overlapping genetic pathways regulated by these factors in the ganglionic eminences [25,26]. Such studies using mice harboring mutations at multiple loci provide great insight into common and distinct functions of transcriptional regulators and their downstream actions.

## Genetic pathways to MGE-derived cortical interneuron fates

The MGE is the largest source of interneurons for the cortex, generating around 60% of the total population [4,5^••^,6,7^••^,8]. This includes two major classes: firstly, parvalbumin (PV)-expressing, fast spiking basket and Chandelier cells and secondly, somatostatin (SST)-expressing neurons that may express other markers such as calretinin (CR), neuropeptide Y (NPY) or reelin (RLN), may have multipolar, bitufted or bipolar dendrites, distinct axonal arborizations and may exhibit intrinsic-burst spiking or adapting non-fast spiking responses to current injection (Figure 1) [12]. Although lumped into two classes, PV-expressing and SST-expressing interneurons are themselves diverse populations. What are the molecular pathways that direct their fates? And by fate we refer to molecular identity, laminar localization, axonal/dendritic morphology and physiological characteristics, all of which are used as traits for classification.

At the top of the molecular hierarchy governing MGE-interneuron development is NKX2-1 (Table 1 and Figure 2). The actions of NKX2-1 are central to the MGE and are initiated through specification of the neuroepithelial MGE identity [4,5^••^,6,7^••^,8]. In its absence, interneurons known to be derived from this region are mis-specified into alternative fates. Yet *Nkx2-1* is only briefly expressed in the cortical interneuron lineage and becomes downregulated in migrating immature cells as part of their differentiation program [4,5^••^,6,7^••^,8].

ZEB2 (also referred to as SIP1) has recently been identified as another direct target of DLX1/2 [27^•^]. Although its functions have been characterized in the MGE, ZEB2 may also play a role in the CGE-derived cortical interneuron lineage [28^•^]. In ZEB2 conditional LOF mutants MGE interneurons fail to migrate to the cortex due to upregulation of the guidance receptor UNC5B [28^•^]. ZEB2-deficient MGE-derived cells remain instead in the subpallium and switch to a striatal fate [27^•^]. This suggested that ZEB2 may act as a fate-determining factor in the MGE regulating cortical versus subcortical interneuron fate [27^•^]. The alternative possibility that the fate-switch may be due to cell non-autonomous defects occurring from exposure to an ectopic environment remains to be explored.

LHX6 is a transcription factor that is directly activated by NKX2-1 and has a central role in neuronal development from the MGE. Within the cortical interneuron lineage LHX6 is required for migration, correct laminar distribution and normal differentiation of PV-expressing and SST-expressing cortical interneurons [4,5^••^,6,7^••^,8]. Intriguing findings in hypomorphic LHX6 mutants where SST^+ve^ but not the PV^+ve^ interneurons are affected suggest that the two populations may have different dose requirements for LHX6 for their normal development [29]. This idea casts further light onto the mechanism of SST versus PV fate-specification from MGE precursors (see subsequent sections).

SOX6 has been identified as acting downstream of LHX6 in immature MGE-derived cortical interneurons. LOF studies in mice have revealed that SOX6 is not involved in specification of subtype identity but is essential for correct laminar position and maturation within the network [4,5^••^,6,7^••^,8]. The extent to which the maturation defects can be attributed to mis-positioning and lack of normal wiring partners or to cell-autonomous loss of SOX6 is unclear. Intriguingly, even though SOX6-deficient interneurons are mis-positioned within the cortex they still wire up to the cortical network indicating some degree of plasticity in synaptic partner selection.

The DLX transcriptional regulators have additional functions beyond the initial specification and migration stages. DLX1 is required for dendritic maturation and survival of SST^+ve^, NPY^+ve^ and CR^+ve^ interneurons whereas DLX5/6 are required for development of PV^+ve^ interneurons [4,5^••^,6,7^••^,8]. On the basis of these findings it has been suggested that LHX6 may act together with either DLX1 to promote the SST fate or DLX5/6 to promote the PV fate [30].

Other transcription factor-encoding genes have been identified in MGE-derived cortical interneurons through expression profiling and other studies. Examples include *Cux2*, *Nr4a1*, *Rora*, *Mef2c*, *MafB* and its relative *cMaf* [31–33]. For some of these genes validation of expression is still pending, and their significance in cortical interneuron development awaits confirmation by functional studies.

## Specifying the dMGE fate

Genetic fate-mapping has shown that cortical Martinotti cells co-expressing SST and CR originate exclusively from the dMGE indicating that this region has unique differentiation potential (Figure 1) [4,5^••^,6,7^••^,8]. How is this achieved? The dMGE is morphologically continuous but molecularly distinct from the rest of the MGE as it expresses markers such as *Gli1* and *Nkx6-2* (Table 1 and Figure 2). Since the expression of these two transcription factors is usually associated with high levels of SHH signaling, it has been proposed that the dMGE is specified by increased exposure to SHH. The findings that the dMGE has a bias for generating SST over PV cortical interneurons and that high SHH signaling promotes SST over PV fate support the notion of dMGE fate-specification through enhanced SHH signals [4,5^••^,6,7^••^,8,15].

More recent work into the function of LHX6 and its related factor LHX8 (also referred to as LHX7) has once more demonstrated the requirement for high SHH signals in the development of the dMGE [34]. According to the proposed model, LHX6 and LHX8, which are expressed in postmitotic neurons in the developing MGE, promote activation of the *Shh* gene in the same cells. SHH secreted from these neurons feeds forward onto the overlying VZ of the MGE to specify dMGE fates by promoting upregulation of *Gli1*, *Nkx2-1*, *Ptch1* and *Nkx6-2* [34]. Expression of some of these genes is restricted to the dMGE thus endowing this region with its distinct identity. The demonstration that certain interneuron subtypes are generated exclusively from the dMGE shows once more how molecular subdivision of neuroepithelial precursors specifies mature neuronal fates.

## Opening the black box of CGE interneuron fate specification

The CGE is the second largest contributor to cortical and hippocampal interneurons generating 30–40% of the total population in the adult cortex (Figure 1) [4,5^••^,6,7^••^,8]. Interneurons generated from the dorsal CGE (dCGE) are distinct from those of the MGE and include two major classes: firstly, RLN-expressing (SST^−ve^) late-spiking cells that have multipolar morphology and secondly, vasoactive intestinal peptide (VIP)-expressing irregular-spiking or fast-adapting cells that may co-express CR and may have bipolar/bitufted or sometimes multipolar morphologies (Figure 1) [12]. Knowledge of the genetic pathways that specify these fates is only now beginning to emerge partly because of the paucity of tools that could be used to uniquely label the CGE and its neuronal progeny.

At the top of the hierarchy governing the development of the CGE and its rostral extension, the lateral ganglionic eminence (LGE), is the transcription factor GSX2 (also referred to as GSH2) (Table 1 and Figure 2) [4,5^••^,6,7^••^,8]. GSX2 is enriched in (but not restricted to) the neuroepithelium of the LGE/CGE from early development. Its initial function is to promote expression of downstream genes such as ASCL1, DLX2 and OLIG2-factors that initiate different aspects of LGE/CGE identity [35]. In addition, GSX2 has been directly implicated in promoting the CR-expressing interneuron identity [36]. A related gene, GSX1, is co-expressed with GSX2 in the ventral telencephalon. The two have common functions in the specification of LGE/CGE identity but differentially regulate neurogenesis with GSX2 maintaining a progenitor state and GSX1 promoting neuronal differentiation [37^•^].

Another three transcription factors that have been implicated in CGE-derived interneuron development are NR2F1, NR2F2 and SP8. Expression of NR2F1 (also referred to as COUPTFI) is enriched in the CGE, the dMGE and POA but it is not restricted to these regions [38]. A role for NR2F1 in interneuron development has been demonstrated through conditional LOF studies which resulted in an imbalance of interneuron subtypes in the cortex [38]. This has been attributed to a defect in progenitor proliferation rather than cell fate determination [38]. The related transcription factor, NR2F2 (COUPTFII), is involved in directing interneurons through a caudal migration route [4,5^••^,6,7^••^,8]. Like NR2F1, expression of NR2F2 is not linked to a single origin and can be observed in MGE-derived as well as CGE-derived interneurons [4,5^••^,6,7^••^,8,39]. More recently, SP8 has been identified as a marker for some CGE interneurons; its function in the lineage remains unknown [40^•^].

A breakthrough into the specification of CGE fates has been the finding of PROX1 expression in the lineage. PROX1 is a transcription factor that is present in nearly all striatal interneurons regardless of their origin but within the cortical interneuron population expression is confined to CGE and POA-derived cells [41^•^]. LOF studies in mice have demonstrated an essential role for this transcription factor in the development of CGE-derived cortical interneurons: at early stages PROX1 is necessary for radial migration and proper positioning within the cortical plate; at later stages the requirement for PROX1 is subtype-specific, functioning in morphogenesis, maturation and network integration (G Miyoshi and G Fishell, personal communication). CGE-derived interneurons lacking PROX1 maintain expression of NR2F2 and SP8 suggesting independent activation of these two transcription factors (G Miyoshi and G Fishell, personal communication). PROX1 is therefore a lineage tracer for the CGE-derived cortical interneuron population acting at multiple points to regulate their differentiation. How a single transcription factor such as PROX1 (or LHX6 in the MGE-lineage) can have multiple functions in different cell types and at different stages of development is unknown but likely to be mediated by differential binding to as yet unidentified transcriptional cofactors.

## The mysterious POA-derived interneurons

Interneurons generated from the POA contribute only ∼10% of the total population in the adult cortex but include a large diversity of subtypes (Figure 1) [4,5^••^,6,7^••^,8]. As the POA has only recently been placed on the source map of cortical interneurons we have almost no data on how these cells are specified. Genes involved in fate-direction elsewhere in the telencephalon are also expressed in the POA and contribute to patterning of this domain (Figure 2). These include SHH and NKX2-1 which are expressed in the majority of the POA neuroepithelium, DBX1 and NKX6-2 which label respectively the dorsal and ventral POA domains and the postmitotic marker HMX3 (also referred to as NKX5-1), which is expressed in small subsets of cells adjacent to the neuroepithelium [14,42,43]. Some of these genes have been used in lineage tracing studies of the POA [42,43] but their contribution to interneuron specification remains elusive.

## Genetic pathways and environmental cues: nature and nurture

There are numerous overlapping steps in cortical interneuron development before a fully mature phenotype is established. These include tangential migration through the subpallium and the pallium, radial migration and layer selection within the cortical plate, formation of axonal and dendritic arborizations, expression of mature markers related to physiological properties, synaptic target cell selection and subcellular targeting of synapses (Figure 2). There is evidence showing that nearly all of these are linked to the embryonic origin of interneurons and therefore are specified by genetic pathways. Even cell death, a process by which 40% of interneurons generated during development are eliminated, is thought to be determined by intrinsic factors [44]. However, genetic programs do not act in isolation and environmental cues are essential for their correct execution. For example, from the onset of their migratory journey, interneurons depend on guidance cues secreted by the environment to find their way to their destination. In the absence of such signals interneuron distribution becomes abnormal [45]. Late-born CR-expressing interneurons additionally require electrical activity for migration as well as development of their axonal and dendritic arbors [46^••^]. Furthermore, layer acquisition and connectivity, both of which show high specificity, are determined by embryonic origin but are also dependent on local cues [47^••^,48,49,50^••^]. And even expression of neurotransmitters, channels and neurotransmitter receptors is genetically predetermined but requires external influences for acquisition of mature phenotypic features [51,52^•^].

The discovery of the activity-dependent expression of SATB1 in cortical interneurons is one of the most recent examples of environmental influences on the genetic program of interneuron development [53^•^,54^•^]. SATB1 is a maturation-promoting factor that is expressed in subsets of cortical interneurons. In its absence, SST-expressing interneurons lose hallmarks of their identity [53^•^,54^•^]. They do not convert to an alternative fate but simply remain as immature neurons. Expression of SATB1 is detected just before birth and evidence suggests that this is dependent on cortical activity [53^•^,54^•^]. Yet induction of SATB1 is restricted to MGE interneurons and requires LHX6 function [54^•^]. SATB1 therefore forms the link between a developmentally imposed genetic specification program and extrinsic environmental cues; a prime example of nature and nurture intertwined to specify cell fate.

## Concluding remarks

We currently have a framework of the initial genetic pathways that lead to cortical interneuron cell fates but we are far from a complete picture (Figure 2). We lack almost any insight into late developmental events such as specification of axonal and dendritic blueprints, synaptic partner selection or expression of channels and receptors that define the physiological characteristics of mature interneurons. These processes are all likely to be highly dependent on intrinsic factors and environmental influences. Some of the early-acting genes already identified are undoubtedly acting as ‘master’ regulators that trigger downstream genetic cascades. As new factors come into play these will either feed into the known pathways or expand the branches to further refine our understanding of the mechanisms that control cortical interneuron trait acquisition.

## References and recommended reading

Papers of particular interest, published within the period of review, have been highlighted as:

• of special interest
•• of outstanding interest

## Figures and Tables

**Table 1:** Transcription factors and reported functions in cortical interneuron development

Transcription factor	Function in cortical interneuron development	References^a^	Association with human psychiatric/neurological disorders^b^
ARX	Migration	[55,56]	X-Linked Mental Retardation; X-Linked Lissencephaly with Abnormal Genitalia (mutations); Proud syndrome; Partington syndrome; West syndrome (mutations)
ASCL1 (MASH1)	Neuroepithelial patterning, neurogenesis	[26,57,58]	
DBX1	Unknown	[42]	
DLX1/2	Inhibition of glial fate, promotion of GABAergic phenotype, migration, differentiation, survival	[26,59–61]	Autism Spectrum Disorder (SNP association)
DLX5/6	Migration, differentiation	[30]	Autism Spectrum Disorder (mutation); Rett Syndrome (epigenetic)
GLI1	Neuroepithelial patterning	[36]	
GSX1/2	Neuroepithelial patterning, neurogenesis, cell fate	[35,36,37^•^]	
HMX3 (NKX5.1)	Unknown	[43]	
LHX6	Migration, laminar distribution, differentiation	[29,33,34,62]	Schizophrenia (low *Lhx6* RNA expression in some patients)
NKX2-1	Neuroepithelial patterning, cell fate, migration	[63–65]	
NKX6-2	Neuroepithelial patterning, cell fate	[36,66,67]	
NR2F1 (COUPTFI)	Progenitor proliferation	[38]	
NR2F2 (COUPTFII)	Migration	[68]	
PROX1	Migration, differentiation, maturation	[41^•^], Miyoshi and Fishell, personal communication	
SATB1	Maturation	[53^•^,54^•^]	
SOX6	Laminar distribution, maturation	[69,70]	
SP8	Unknown	[40^•^]	Bipolar Disorder (locus and intergenic SNP association); Schizophrenia (locus association); Psychosis (locus association)
ZEB2 (SIP1)	Cell fate, migration,	[27^•^,28^•^]	Mowat–Wilson syndrome

a Literature describing mouse mutants and/or other studies that provide insight into function in cortical interneuron development.

b Association of transcription factors with human psychiatric/neurological disorders reported in the OMIM (Online Mendelian Inheritance in Man), GAD (Genetic Association Database) and Disgenet databases.

## References

[1] Klausberger T., Somogyi P. (2008). Neuronal diversity and temporal dynamics: the unity of hippocampal circuit operations. Science.

[2] Ascoli G.A., Alonso-Nanclares L., Anderson S.A., Barrionuevo G., Benavides-Piccione R., Burkhalter A., Buzsaki G., Cauli B., DeFelipe J., Fairen A. (2008). Petilla terminology: nomenclature of features of GABAergic interneurons of the cerebral cortex. Nat Rev Neurosci.

[3••] DeFelipe J., Lopez-Cruz P.L., Benavides-Piccione R., Bielza C., Larranaga P., Anderson S., Burkhalter A., Cauli B., Fairen A., Feldmeyer D. (2013). New insights into the classification and nomenclature of cortical GABAergic interneurons. Nat Rev Neurosci.

[4] Anastasiades P.G., Butt S.J. (2011). Decoding the transcriptional basis for GABAergic interneuron diversity in the mouse neocortex. Eur J Neurosci.

[5••] Gelman D.M., Marin O., Rubenstein J.L.R., Noebels J.L., Avoli M., Rogawski M.A., Olsen R.W., Delgado-Escueta A.V. (2012). The generation of cortical interneurons. Jasper's Basic Mechanisms of the Epilepsies [Internet].

[6] Hernandez-Miranda L.R., Parnavelas J.G., Chiara F. (2010). Molecules and mechanisms involved in the generation and migration of cortical interneurons. ASN Neuro.

[7••] Miyoshi G., Machold R.P., Fishell G. (2013). Specification of GABAergic neocortical interneurons. Cortical Development: Neural Diversity and Neocortical Organization.

[8] Wonders C.P., Anderson S.A. (2006). The origin and specification of cortical interneurons. Nat Rev Neurosci.

[9] Cambray S., Arber C., Little G., Dougalis A.G., de Paola V., Ungless M.A., Li M., Rodriguez T.A. (2012). Activin induces cortical interneuron identity and differentiation in embryonic stem cell-derived telencephalic neural precursors. Nat Commun.

[10••] Marin O. (2012). Interneuron dysfunction in psychiatric disorders. Nat Rev Neurosci.

[11] Maroof A.M., Keros S., Tyson J.A., Ying S.W., Ganat Y.M., Merkle F.T., Liu B., Goulburn A., Stanley E.G., Elefanty A.G., Widmer H.R., Eggan K., Goldstein P.A., Anderson S.A., Studer L. (2013). Directed differentiation and functional maturation of cortical interneurons from human embryonic stem cells. Cell Stem Cell.

[12] Fishell G., Rudy B. (2011). Mechanisms of inhibition within the telencephalon: “where the wild things are”. Annu Rev Neurosci.

[13] Rubin A.N., Alfonsi F., Humphreys M.P., Choi C.K., Rocha S.F., Kessaris N. (2010). The germinal zones of the basal ganglia but not the septum generate GABAergic interneurons for the cortex. J Neurosci.

[14] Flames N., Pla R., Gelman D.M., Rubenstein J.L., Puelles L., Marin O. (2007). Delineation of multiple subpallial progenitor domains by the combinatorial expression of transcriptional codes. J Neurosci.

[15] Inan M., Welagen J., Anderson S.A. (2012). Spatial and temporal bias in the mitotic origins of somatostatin- and parvalbumin-expressing interneuron subgroups and the chandelier subtype in the medial ganglionic eminence. Cereb Cortex.

[16] Miyoshi G., Butt S.J., Takebayashi H., Fishell G. (2007). Physiologically distinct temporal cohorts of cortical interneurons arise from telencephalic Olig2-expressing precursors. J Neurosci.

[17••] Taniguchi H., Lu J., Huang Z.J. (2013). The spatial and temporal origin of Chandelier cells in mouse neocortex. Science.

[18••] Ciceri G., Dehorter N., Sols I., Huang Z.J., Maravall M., Marin O. (2013). Lineage-specific laminar organization of cortical GABAergic interneurons. Nat Neurosci.

[19] Franco S.J., Gil-Sanz C., Martinez-Garay I., Espinosa A., Harkins-Perry S.R., Ramos C., Muller U. (2012). Fate-restricted neural progenitors in the mammalian cerebral cortex. Science.

[20] Cohen M., Briscoe J., Blassberg R. (2013). Morphogen interpretation: the transcriptional logic of neural tube patterning. Curr Opin Genet Dev.

[21] Hebert J.M., Fishell G. (2008). The genetics of early telencephalon patterning: some assembly required. Nat Rev Neurosci.

[22] Hoch R.V., Rubenstein J.L., Pleasure S. (2009). Genes and signaling events that establish regional patterning of the mammalian forebrain. Semin Cell Dev Biol.

[23] Schuurmans C., Guillemot F. (2002). Molecular mechanisms underlying cell fate specification in the developing telencephalon. Curr Opin Neurobiol.

[24] Sousa V.H., Fishell G. (2010). Sonic hedgehog functions through dynamic changes in temporal competence in the developing forebrain. Curr Opin Genet Dev.

[25] Long J.E., Swan C., Liang W.S., Cobos I., Potter G.B., Rubenstein J.L. (2009). Dlx1&2 and Mash1 transcription factors control striatal patterning and differentiation through parallel and overlapping pathways. J Comp Neurol.

[26] Long J.E., Cobos I., Potter G.B., Rubenstein J.L. (2009). Dlx1&2 and Mash1 transcription factors control MGE and CGE patterning and differentiation through parallel and overlapping pathways. Cereb Cortex.

[27•] McKinsey G.L., Lindtner S., Trzcinski B., Visel A., Pennacchio L.A., Huylebroeck D., Higashi Y., Rubenstein J.L. (2013). Dlx1&2-dependent expression of Zfhx1b (Sip1, Zeb2) regulates the fate switch between cortical and striatal interneurons. Neuron.

[28•] van den Berghe V., Stappers E., Vandesande B., Dimidschstein J., Kroes R., Francis A., Conidi A., Lesage F., Dries R., Cazzola S. (2013). Directed migration of cortical interneurons depends on the cell-autonomous action of Sip1. Neuron.

[29] Neves G., Shah M.M., Liodis P., Achimastou A., Denaxa M., Roalfe G., Sesay A., Walker M.C., Pachnis V. (2013). The LIM homeodomain protein Lhx6 regulates maturation of interneurons and network excitability in the mammalian cortex. Cereb Cortex.

[30] Wang Y., Dye C.A., Sohal V., Long J.E., Estrada R.C., Roztocil T., Lufkin T., Deisseroth K., Baraban S.C., Rubenstein J.L. (2010). Dlx5 and Dlx6 regulate the development of parvalbumin-expressing cortical interneurons. J Neurosci.

[31] Cobos I., Long J.E., Thwin M.T., Rubenstein J.L. (2006). Cellular patterns of transcription factor expression in developing cortical interneurons. Cereb Cortex.

[32] Faux C., Rakic S., Andrews W., Yanagawa Y., Obata K., Parnavelas J.G. (2010). Differential gene expression in migrating cortical interneurons during mouse forebrain development. J Comp Neurol.

[33] Zhao Y., Flandin P., Long J.E., Cuesta M.D., Westphal H., Rubenstein J.L. (2008). Distinct molecular pathways for development of telencephalic interneuron subtypes revealed through analysis of Lhx6 mutants. J Comp Neurol.

[34] Flandin P., Zhao Y., Vogt D., Jeong J., Long J., Potter G., Westphal H., Rubenstein J.L. (2011). Lhx6 and Lhx8 coordinately induce neuronal expression of Shh that controls the generation of interneuron progenitors. Neuron.

[35] Wang B., Long J.E., Flandin P., Pla R., Waclaw R.R., Campbell K., Rubenstein J.L. (2013). Loss of Gsx1 and Gsx2 function rescues distinct phenotypes in Dlx1/2 mutants. J Comp Neurol.

[36] Xu Q., Guo L., Moore H., Waclaw R.R., Campbell K., Anderson S.A. (2010). Sonic hedgehog signaling confers ventral telencephalic progenitors with distinct cortical interneuron fates. Neuron.

[37•] Pei Z., Wang B., Chen G., Nagao M., Nakafuku M., Campbell K. (2011). Homeobox genes Gsx1 and Gsx2 differentially regulate telencephalic progenitor maturation. Proc Natl Acad Sci U S A.

[38] Lodato S., Tomassy G.S., De Leonibus E., Uzcategui Y.G., Andolfi G., Armentano M., Touzot A., Gaztelu J.M., Arlotta P., de la Menendez P. (2011). Loss of COUP-TFI alters the balance between caudal ganglionic eminence- and medial ganglionic eminence-derived cortical interneurons and results in resistance to epilepsy. J Neurosci.

[39] Cai Y., Zhang Q., Wang C., Zhang Y., Ma T., Zhou X., Tian M., Rubenstein J.L., Yang Z. (2013). Nuclear receptor COUP-TFII-expressing neocortical interneurons are derived from the medial and lateral/caudal ganglionic eminence and define specific subsets of mature interneurons. J Comp Neurol.

[40•] Ma T., Zhang Q., Cai Y., You Y., Rubenstein J.L., Yang Z. (2012). A subpopulation of dorsal lateral/caudal ganglionic eminence-derived neocortical interneurons expresses the transcription factor Sp8. Cereb Cortex.

[41•] Rubin A.N., Kessaris N. (2013). PROX1: a lineage tracer for cortical interneurons originating in the lateral/caudal ganglionic eminence and preoptic area. PLoS ONE.

[42] Gelman D., Griveau A., Dehorter N., Teissier A., Varela C., Pla R., Pierani A., Marin O. (2011). A wide diversity of cortical GABAergic interneurons derives from the embryonic preoptic area. J Neurosci.

[43] Gelman D.M., Martini F.J., Nobrega-Pereira S., Pierani A., Kessaris N., Marin O. (2009). The embryonic preoptic area is a novel source of cortical GABAergic interneurons. J Neurosci.

[44] Southwell D.G., Paredes M.F., Galvao R.P., Jones D.L., Froemke R.C., Sebe J.Y., Alfaro-Cervello C., Tang Y., Garcia-Verdugo J.M., Rubenstein J.L. (2012). Intrinsically determined cell death of developing cortical interneurons. Nature.

[45] Marin O. (2013). Cellular and molecular mechanisms controlling the migration of neocortical interneurons. Eur J Neurosci.

[46••] De Marco Garcia N.V., Karayannis T., Fishell G. (2011). Neuronal activity is required for the development of specific cortical interneuron subtypes. Nature.

[47••] Bartolini G., Ciceri G., Marin O. (2013). Integration of GABAergic interneurons into cortical cell assemblies: lessons from embryos and adults. Neuron.

[48] Brown K.N., Chen S., Han Z., Lu C.H., Tan X., Zhang X.J., Ding L., Lopez-Cruz A., Saur D., Anderson S.A., Huang K., Shi S.H. (2011). Clonal production and organization of inhibitory interneurons in the neocortex. Science.

[49] Lodato S., Rouaux C., Quast K.B., Jantrachotechatchawan C., Studer M., Hensch T.K., Arlotta P. (2011). Excitatory projection neuron subtypes control the distribution of local inhibitory interneurons in the cerebral cortex. Neuron.

[50••] Pfeffer C.K., Xue M., He M., Huang Z.J., Scanziani M. (2013). Inhibition of inhibition in visual cortex: the logic of connections between molecularly distinct interneurons. Nat Neurosci.

[51] Batista-Brito R., Machold R., Klein C., Fishell G. (2008). Gene expression in cortical interneuron precursors is prescient of their mature function. Cereb Cortex.

[52•] Matta J.A., Pelkey K.A., Craig M.T., Chittajallu R., Jeffries B.W., McBain C.J. (2013). Developmental origin dictates interneuron AMPA and NMDA receptor subunit composition and plasticity. Nat Neurosci.

[53•] Close J., Xu H., De Marco G.N., Batista-Brito R., Rossignol E., Rudy B., Fishell G. (2012). Satb1 is an activity-modulated transcription factor required for the terminal differentiation and connectivity of medial ganglionic eminence-derived cortical interneurons. J Neurosci.

[54•] Denaxa M., Kalaitzidou M., Garefalaki A., Achimastou A., Lasrado R., Maes T., Pachnis V. (2012). Maturation-promoting activity of SATB1 in MGE-derived cortical interneurons. Cell Rep.

[55] Colombo E., Collombat P., Colasante G., Bianchi M., Long J., Mansouri A., Rubenstein J.L., Broccoli V. (2007). Inactivation of Arx, the murine ortholog of the X-linked lissencephaly with ambiguous genitalia gene, leads to severe disorganization of the ventral telencephalon with impaired neuronal migration and differentiation. J Neurosci.

[56] Kitamura K., Yanazawa M., Sugiyama N., Miura H., Iizuka-Kogo A., Kusaka M., Omichi K., Suzuki R., Kato-Fukui Y., Kamiirisa K. (2002). Mutation of ARX causes abnormal development of forebrain and testes in mice and X-linked lissencephaly with abnormal genitalia in humans. Nat Genet.

[57] Casarosa S., Fode C., Guillemot F. (1999). Mash1 regulates neurogenesis in the ventral telencephalon. Development.

[58] Castro D.S., Martynoga B., Parras C., Ramesh V., Pacary E., Johnston C., Drechsel D., Lebel-Potter M., Garcia L.G., Hunt C. (2011). A novel function of the proneural factor Ascl1 in progenitor proliferation identified by genome-wide characterization of its targets. Genes Dev.

[59] Anderson S.A., Eisenstat D.D., Shi L., Rubenstein J.L. (1997). Interneuron migration from basal forebrain to neocortex: dependence on Dlx genes. Science.

[60] Cobos I., Calcagnotto M.E., Vilaythong A.J., Thwin M.T., Noebels J.L., Baraban S.C., Rubenstein J.L. (2005). Mice lacking Dlx1 show subtype-specific loss of interneurons, reduced inhibition and epilepsy. Nat Neurosci.

[61] Petryniak M.A., Potter G.B., Rowitch D.H., Rubenstein J.L. (2007). Dlx1 and Dlx2 control neuronal versus oligodendroglial cell fate acquisition in the developing forebrain. Neuron.

[62] Liodis P., Denaxa M., Grigoriou M., Akufo-Addo C., Yanagawa Y., Pachnis V. (2007). Lhx6 activity is required for the normal migration and specification of cortical interneuron subtypes. J Neurosci.

[63] Butt S.J., Sousa V.H., Fuccillo M.V., Hjerling-Leffler J., Miyoshi G., Kimura S., Fishell G. (2008). The requirement of Nkx2-1 in the temporal specification of cortical interneuron subtypes. Neuron.

[64] Nobrega-Pereira S., Kessaris N., Du T., Kimura S., Anderson S.A., Marin O. (2008). Postmitotic Nkx2-1 controls the migration of telencephalic interneurons by direct repression of guidance receptors. Neuron.

[65] Sussel L., Marin O., Kimura S., Rubenstein J.L. (1999). Loss of Nkx2.1 homeobox gene function results in a ventral to dorsal molecular respecification within the basal telencephalon: evidence for a transformation of the pallidum into the striatum. Development.

[66] Fogarty M., Grist M., Gelman D., Marin O., Pachnis V., Kessaris N. (2007). Spatial genetic patterning of the embryonic neuroepithelium generates GABAergic interneuron diversity in the adult cortex. J Neurosci.

[67] Sousa V.H., Miyoshi G., Hjerling-Leffler J., Karayannis T., Fishell G. (2009). Characterization of Nkx6-2-derived neocortical interneuron lineages. Cereb Cortex.

[68] Kanatani S., Yozu M., Tabata H., Nakajima K. (2008). COUP-TFII is preferentially expressed in the caudal ganglionic eminence and is involved in the caudal migratory stream. J Neurosci.

[69] Azim E., Jabaudon D., Fame R.M., Macklis J.D. (2009). SOX6 controls dorsal progenitor identity and interneuron diversity during neocortical development. Nat Neurosci.

[70] Batista-Brito R., Rossignol E., Hjerling-Leffler J., Denaxa M., Wegner M., Lefebvre V., Pachnis V., Fishell G. (2009). The cell-intrinsic requirement of Sox6 for cortical interneuron development. Neuron.

